# Serum Protein Signatures Differentiating Autoimmune Pancreatitis versus Pancreatic Cancer

**DOI:** 10.1371/journal.pone.0082755

**Published:** 2013-12-09

**Authors:** Klaus Felix, Oliver Hauck, Stefan Fritz, Ulf Hinz, Martina Schnölzer, Tore Kempf, Uwe Warnken, Angelika Michel, Michael Pawlita, Jens Werner

**Affiliations:** 1 Department of General Surgery, University of Heidelberg, INF 110, Heidelberg, Germany; 2 Functional Proteome Analysis, German Cancer Research Center (DKFZ), INF 580, Heidelberg, Germany; 3 Infection and Cancer Program, German Cancer Research Center (DKFZ), INF 260, Heidelberg, Germany; Klinikum rechts der Isar der TU München, Germany

## Abstract

Autoimmune pancreatitis (AIP) is defined by characteristic lymphoplasmacytic infiltrate, ductal strictures and a pancreatic enlargement or mass that can mimic pancreatic cancer (PaCa). The distinction between this benign disease and pancreatic cancer can be challenging. However, an accurate diagnosis may pre-empt the misdiagnosis of cancer, allowing the appropriate medical treatment of AIP and, consequently, decreasing the number of unnecessary pancreatic resections.

Mass spectrometry (MS) and two-dimensional differential gel electrophoresis (2D-DIGE) have been applied to analyse serum protein alterations associated with AIP and PaCa, and to identify protein signatures indicative of the diseases. Patients' sera were immunodepleted from the 20 most prominent serum proteins prior to further 2D-DIGE and image analysis. The identity of the most-discriminatory proteins detected, was performed by MS and ELISAs were applied to confirm their expression. Serum profiling data analysis with 2D-DIGE revealed 39 protein peaks able to discriminate between AIP and PaCa. Proteins were purified and further analysed by MALDI-TOF-MS. Peptide mass fingerprinting led to identification of eleven proteins. Among them apolipoprotein A-I, apolipoprotein A-II, transthyretin, and tetranectin were identified and found as 3.0-, 3.5-, 2-, and 1.6-fold decreased in PaCa sera, respectively, whereas haptoglobin and apolipoprotein E were found to be 3.8- and 1.6-fold elevated in PaCa sera. With the exception of haptoglobin the ELISA results of the identified proteins confirmed the 2D-DIGE image analysis characteristics. Integration of the identified serum proteins as AIP markers may have considerable potential to provide additional information for the diagnosis of AIP to choose the appropriate treatment.

## Introduction

Autoimmune pancreatitis (AIP) is a distinct clinical entity, described as a chronic inflammatory process of the pancreas with autoimmune mechanisms. Clinically and histologically, two subsets of autoimmune pancreatitis (type 1 and type 2 AIP) exist and should be distinguished [1]–[4]. The type 1 AIP, a lymphoplasmacytic sclerosing pancreatitis (LPSP), shows some typical features: periductal lymphoplasmacytic infiltrate, fibrosis, obliterative venulitis, and infiltration of IgG4-positive plasma cells. The type 2 AIP idiopathic duct-centric pancreatitis (IDCP) is characterised by massive infiltration of granulocytes in the pancreatic parenchyma and ductal epithelial lesions (GEL). These features are described in the Mayo HISORt criteria, which we use in our clinic [5]. Type 1 AIP predominantly affects adult males with >90% of patients being more than 40 years of age [6]. The most common clinical presentation of type 1 AIP is acute obstructive jaundice, which is reported in up to 75% of patients [7]. In addition, a pancreatic enlargement or mass can mimic pancreatic cancer in up to 80% of patients [1]. In the presence of a new onset of diabetes and weight loss, the distinction between AIP and pancreatic cancer can be challenging. Additionally, on a CT scan or magnetic resonance imaging (MRI), a certain “sausage-shaped” enlargement of the pancreas with delayed peripheral enhancement (rim enhancement) is described [8]–[10]. Endoscopic Retrograde Cholangio-Pancreatography (ERCP) reveals typical segmental narrowing and multiple strictures, which can help to differentiate between pancreatic cancer and primary sclerosing cholangitis [11]–[13]. Type 1 AIP presents several serological characteristics. The most prominent of them is elevated serum levels of IgG4, which is crucial for diagnosis in absence of histology according to the Mayo HISORt criteria [5]. Furthermore, antinuclear antibodies, anticarbonic anhydrase, and antilactoferrin may be increased too. AIP can often be difficult to distinguish from PaCa as the patients' demographics, as well as the clinical and imaging features (e.g. pancreatic enlargement, obstructive jaundice in 76%, weight loss in 35% of patients), are similar. Therefore, it is desirable to recognise AIP since 2.5–11% of all patients undergoing surgery for suspected PaCa are actually having a benign inflammatory disease of the pancreas [14]–[16]. AIP can be treated by steroids, and the high response to this therapy is an important diagnostic criterion. Therefore, it is extremely important to diagnose AIP to choose the appropriate treatment and avoid unnecessary surgery.

**Figure 1:**
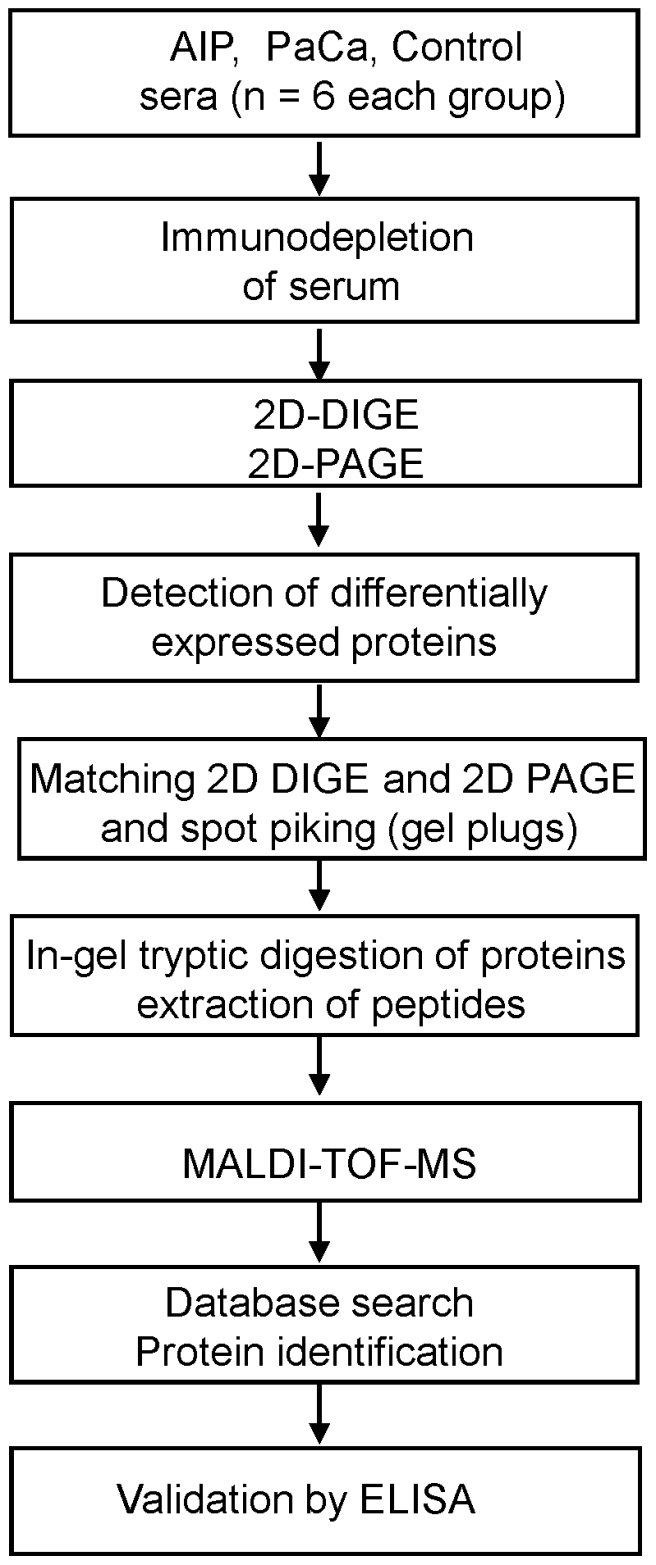
Workflow of the serum proteome analysis for identification of differentially expressed proteins in AIP and PaCa patients. Six sera of each group (AIP, PaCa and Ctr) were first subjected to a reduction of serum complexity by removal of the 20 most abundant serum proteins by immunodepletion columns. The immunoaffinity-processed sera were subjected in parallel to 2D-DIGE and 2 D PAGE, and differentially expressed proteins identified through DIGE image analysis were matched with the preparative gel, The protein spots were excised as gel plugs, prepared for tryptic digestion and identified by MS. The conformation and pilot validation of identified proteins was performed by ELISAs.

The aim of this initial study was to identify serum proteins (serum biomarkers) which allow discriminating AIP from PaCa. For this purpose we applied a proteomic strategy as outlined in figure 1. The identity of the proteins detected was determined by a combination of several techniques, including serum protein fractionation by immunoaffinity substraction of prominent proteins, 2D-gel electrophoresis, and mass spectrometry and finally confirmed and assessed the serum protein levels by enzyme linked immunosorbent assays (ELISA).

## Materials and Methods

### Sample collection

Pancreatic tissue samples were prospectively collected between January 2003 and March 2010 at the Department of Surgery, University of Heidelberg. The diagnosis of chronic pancreatitis (CP), PaCa or AIP was confirmed by histopathology. In case of AIP, the Mayo Clinic (HISORt) criteria were used [5]. The histological examination of formalin-fixed, paraffin-embedded and H&E-stained pancreatic tissue sections was performed by a pathologist at the Pathology Institute, University of Heidelberg.

Preoperative blood was collected from patients diagnosed with adenocarcinoma of the pancreas (PaCa, 270 samples), alcoholic chronic pancreatitis (CP, 290 samples), autoimmune pancreatitis (AIP, 32 samples), a control group of healthy volunteers (Co, 127 samples), and other gastrointestinal cancers (GICa, 165 samples). On admission specific patient characteristics (age, gender, body mass index (BMI) clinical symptoms, weight loss, other organ involvement, complications, blood tests, and treatments, etc.) were documented. All sera were obtained according to a standardised sampling and coding protocol [17].

Ethics Statement: The study was approved by the ethics committee of the University of Heidelberg, and a written informed consent was obtained from all patients.

### Detection of total IgG and IgG4 antibodies

One characteristic feature of AIP is the presence of high levels of serum IgG, particularly the IgG4 subclass. To further assess the diagnostic value of total IgG and IgG4 levels in AIP, detection of the antibodies was performed. The total IgG and the IgG4 subset serum levels were determined at the time of diagnosis by nephelometry, using a BNII Nephelometer (Dade Behring) as a standard assay test to quantify serum immunoglobulins. Total IgG and IgG4 serum levels were considered to be elevated when concentration reached values of ≥ 16.0 g/l and ≥ 1.4 g/l respectively [18].

### Immune response to 15 *H. pylori* proteins

Similar to gastric cancer development, a pathogenic role of *H. pylori* infection has been discussed for pancreatic disease [19]. We investigated the association of antibodies to 15 different *H. pylori* proteins and pancreatic disease compared to *H. pylori*-associated gastric disease. We analysed sera from patients with autoimmune pancreatitis (AIP, n = 32), chronic pancreatitis (CP, n = 290) and pancreatic cancer (PaCa, n = 269), and compared to healthy controls (Co, n = 127) and other GI-tract cancers (GICa, n = 165) with *H. pylori* multiplex serology [20] that allows simultaneous detection of antibodies to 15 *H. pylori*-specific antigens (UreA, Catalase, GroEL, NapA, CagA, CagM, Cagδ, HP0231, VacA, HpaA, Cad, HyuA, Omp, HcpC, HP0305). The multiplex method is based on a glutathione S-transferase capture immunosorbent assay combined with fluorescent-bead technology [21], [22]. Recombinant GST-*H. pylori* fusion proteins were used as antigens, loaded and affinity-purified on spectrally distinct glutathione-casein-coupled (GC) fluorescence-labelled polystyrene beads (SeroMap, Luminex, Austin, Texas). Antibodies bound to the beads via the GST-*H. pylori* fusion proteins were stained with biotinylated goat anti-human IgA, IgM, IgG (Dianova, Hamburg, Germany) and the reporter conjugate R-phycoerythrin-labelled streptavidin. A Luminex 100 analyser identified the internal bead colour and, therefore, the antigen carried by the bead. The quantity of bound antibodies was determined as the median reporter fluorescence intensity (MFI) of at least 100 beads per bead set per serum.

For all 15 antigens, antigen-specific cut-off values determined in a validation study [20] were applied from the MFI values of 20 additional sera negative in Helicobacter-R-Biopharm ELISA as mean plus 3 standard deviations excluding positive outliers. Study data was normalised to this previous validation study by dividing the antigen-specific antibody reactivities by the slopes of the regression lines of the data pairs of a panel of 77 quality control sera with a defined *H. pylori* status run in this and the previous validation study. All sera were analysed once at 1∶100 dilution within a single assay day. *H. pylori* sero-positivity was defined as sero-positivity to >3 proteins, which has shown excellent agreement (kappa = 0.70) with commercial serological assay classification [20].

### Proteome analysis

**2D-DIGE** Prior to two-dimensional differentiation analysis of the AIP, PaCa and control sera, a separation step to eliminate the 20 most prominent serum proteins was performed to obtain a better access to the less prominent serum proteins. For this purpose, patients' sera were subjected to an immunodepletion step using the ProteoPrep 20 kit and user guide (Sigma, Deisenhofen, Germany). The depleted and diluted sera were subsequently adjusted to the required protein concentrations of 1.0 mg/ml by the protein precipitation protocol of Wessel and Flügge, and the protein precipitates were reconstituted in lysis buffer (8 M urea, 20 mM Tris, 4% CHAPS, pH 8.5) [23]. Samples used for 2D-DIGE analysis were labelled with CyDye DIGE fluor minimal dyes (GE-Healthcare GmbH, Freiburg, Germany) prior to 2D gel analysis. For this purpose, 50 µg of protein were mixed with 400 pmol CyDye dissolved in DMF, vortexed, spun, and incubated in the dark on ice for 30 min. Excess of dye was bound by adding 1 µl of 10 mM lysine to the mixture. A detailed protocol is described in the User Manual Ettan DIGE System (GE-Healthcare 2005) and [24].

Immobiline DryStrips of 24 cm and pH 3–10 NL (GE Healthcare) were passively loaded with 300 µg of protein samples resuspended in 350 µl in a mixture of 150 µl lysis buffer (7 M urea, 2 M thiourea, 4% w/v CHAPS) and 200 µl destreak solution (2% w/v IPG-ampholyte, 2% w/v dithiotreitol DTT). IEF was performed with an Ettan IPGphor IEF unit system (GE Healthcare) and a voltage gradient of up to 8 kV (total 35.5 kVh). Prior to the second dimension run on a 15% PAGE, the IPG strips were stepwise equilibrated for 15 min in a Tris/HCl-buffer (pH 8.8, 6 M urea, 30% glycerol, 2% SDS) with 1% DTT, followed by 2.5% iodoacetamide. A molecular weight marker (SM0671 Sigma) was used, and the electrophoresis runs over night at constant 12 W. Gels were scanned using a Typhoon 9410 scanner (GE Healthcare). Protein quantitation across all samples was performed using DeCyder 2D 6.5 software (GE Healthcare). Applying a DIA module, the Cy2 labelled internal standard allowed the normalisation of protein spots across all gels. With the BVA module, a Student's t-test (unpaired, two tailed) was used to calculate significant (p<0.05) differences in a relative protein amount/spot in six AIP sera compared to six PaCa sera. Spots there were found to be statistically significant were mapped and isolated for further investigation as described below.

#### Identification of proteins from 2D gels by MALDI-TOF MS

After aligning the silver-stained gels with the corresponding preparative gels, the recurring and matching spots were marked with an ID number. The selected protein spots were punched out from the 2D gels, placed into individual ID-labelled 200 µl tubes, and washed twice with 70% acetonitrile (ACN), 40 mM NH_4_HCO_3_. After reduction with 10 mM DTT for 1 h at 56°C and alkylation with 55 mM iodoacetamide for 30 min at RT, the gel plugs were washed three times alternately with 40 mM NH_4_HCO_3_ and ethanol. After drying with acetonitrile, an in-gel trypsin digestion was performed by adding 100 ng trypsin in 40 mM NH_4_HCO_3_ over night at 37°C. The resulting peptides were extracted from the gel plugs by an addition of 15 µl extraction solution (1% ACN, 1% formic acid).

For mass spectrometric identification of the tryptic digests by peptide mass fingerprinting, samples were prepared on PrespottedAnchorChip (PAC) targets according to the protocol provided by the manufacturer (Bruker-Daltonik, Bremen, Germany). α-cyano-4-hydroxycinnamic acid was used as a matrix [25]. Mass spectra were recorded in the positive ion reflector mode with delayed extraction on an Ultraflex I time-of-flight instrument (Bruker-Daltonik, Bremen, Germany). Ion acceleration was set to 25.0 kV, the reflector to 26.3 kV, and the first extraction plate to 21.75 kV. Mass spectra were obtained by summing up 500–1500 individual laser shots. Calibration of spectra was performed externally by a quadratic fit using a peptide calibration standard mix containing bradykinin (1–7) at m/z 757.399, angiotensin II at m/z 1046.542, angiotensin I at m/z 1296.685, neurotensin at m/z 1672.917, renin substrate at m/z 1758.933, ACTH clip (1–17) at m/z 2093.086, ACTH clip (18–39) at m/z 2465.198, ACTH clip (1–24) at m/z 2932.588, and ACTH clip (7–38) at m/z 3657.929. Singly charged monoisotopic peptide masses were used as inputs for database searching. Searches were performed against the NCBInr database (released 2009-09-09) using the Mascot search algorithm version v2.2 (Matrix Science, London, UK). Taxonomy was mammals, protein mass was restricted to 100 kDa, and isoelectric points were allowed to range from 0 to 14. Carbamidomethylation of cysteine was included as fixed modification, and the oxidation of methionine was allowed as variable modification. Up to one missed tryptic cleavage site was considered, and the mass tolerance for the monoisotopic peptide masses was set to +/−75 ppm.

### Enzyme linked immunosorbent assay (ELISA)

Solid-phase capture sandwich ELISAs were performed using commercially available kits for apolipoprotein A-I (ALerCHEK Inc., Portland, Maine). Apolipoprotein A-II, apolipoprotein E and haptoglobin were from AssayPro (AssayPro, St. Charles, MO), tetranectin from USCN Life Science Inc. (Wuhan, PRC), and transthyretin from Immundiagnostik AG (Bensheim, Germany). The haptoglobin ELISA from AssayPro is also a sandwich ELISA but with a competitive enzyme immunoassay technique. The six used ELISAs quantify the sum of all antigens that bind the antibody and, therefore, do not reveal the relative contribution by isoforms or post-translationally modified forms. All sera were diluted according to the manufacturer's instructions with buffers provided or TBS buffer, and the assays were performed according to the manufacturer's protocols. Carbohydrate antigen 19-9 (CA 19-9) was determined in serum by a sandwich-immunoassay with the use of an electrochemiluminescence immunoassay (ECLIA, Roche Diagnostics, Mannheim, Germany).

### Biometric analysis

Statistical analysis was carried out by GraphPad Prism 5 software (GraphPad software, Inc., San Diego, California, USA) and SAS software release 9.1 (SAS Institute, Cary, North Carolina, USA).

Biometric analysis was performed to examine the strength of correlation between the clinical parameters body-mass-index (BMI) and the tumour marker CA 19-9 with levels of ELISA-based validations of the six protein markers. Depending on the character of the distributions of the quantitative parameters in each group the correlation coefficient r with its corresponding p-value of Pearson and Spearman were used to analyse the correlations. The character of the distributions of the quantitative parameters was determined by using the Shapiro-Wilk test and a normal probability plot.

## Results

### Serological abnormalities

**Figure 2:**
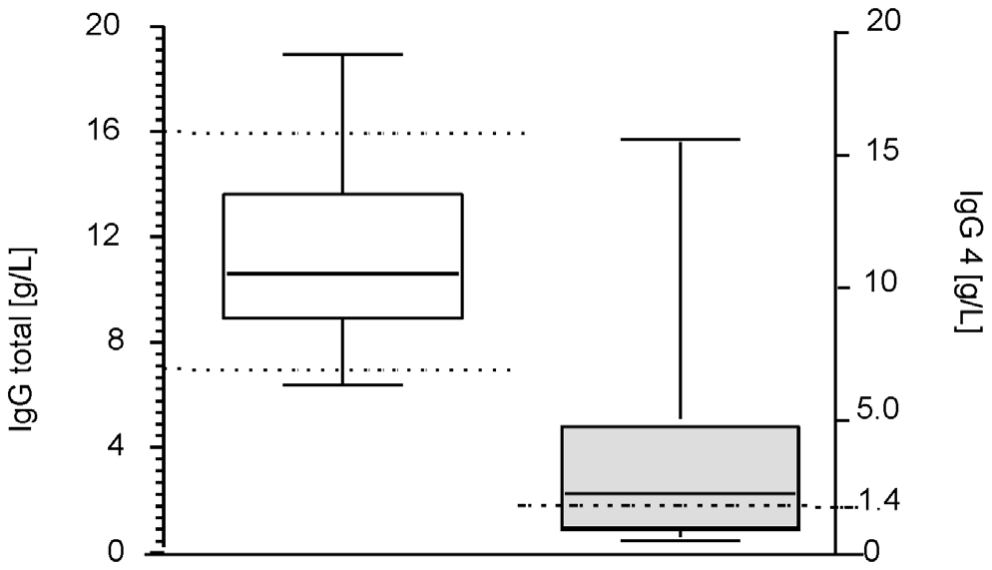
Total IgG and the IgG4 subset serum levels in AIP patients. Serum levels of IgG and IgG4 were determined by automated immunonephelometry, and considered elevated when ≥16.0 g/l and ≥1.4 g/l respectively (dotted lines).

Two clinical patterns are distinguished in patients diagnosed with AIP: classified as type 1 and type 2. We analysed our patient cohort accordingly. A hallmark of AIP type 1 is the elevation of gamma globulin and its subset, the immunoglobulin G4 (IgG4) serum levels; therefore, we analysed our samples for these abnormalities. From the analysed samples (n = 32), three patients were characterised with abnormal total IgG of >16 g/l, and 18 samples (59%) showed elevated IgG4 levels (upper limit 1.4 g/L), ranging between 1.8 and 16.6 g/L (Fig. 2).

### H. pylori and AIP

Since *Helicobacter pylori* infection has been associated with the pathogenesis of AIP, we investigated the association of antibodies to 15 different *H. pylori* proteins in 32 AIP sera and compared to other pancreatic diseases and controls. Antibody analyses were performed with multiplex serology based on recombinantly expressed and affinity-purified *H. pylori* proteins in combination with fluorescent bead (Luminex) technology. *H. pylori* seroprevalence was 43.8% in AIP and 56.9% in CP compared to 64.7% in PaCa, 69.7% in different GI cancers and 50.4% in controls (summarised in Table 1). *H. pylori* seropositive patients with AIP showed significantly lower antibody responses to 3 antigens (Cagδ, CagA, HP0231) and patients with chronic pancreatitis to 9 (Cad, Cagδ, CagA, CagM, HP0305, HpaA, HyuA, Omp, and VacA) compared to *H. pylori* seropositive patients with other gastrointestinal tract cancers as a reference, with there being a significant increase of antibody reactivities to VacA in *H. pylori* seropositive patients with AIP and NapA in those with pancreatic cancer. Among pancreatic disease patients, our data does not support a significant association of *H. pylori* infection with AIP.

**Table 1:** *H. pylori* antibody analyses for pancreatic diseases including AIP and controls with multiplex serology using 15 recombinant, affinity-purified *H. pylori* proteins.

Disease	patients (n)	mean age (SD)	female (%)	*H. pylori* positive (%)
PaCa	269	62.9±10	43.9	64.7
CP	290	50.5±11.7	30.7	56.9
AIP	32	52.2±13.2	3.1	43.8
Healthy controls	127	39.9±15.6	44.9	50.4
Diff. GI cancers	165	59.9±12.5	44.2	69.7

**Serum protein expression profiling applying immunodepletion and 2D-DIGE**: To find and identify potential diagnostic biomarkers for differential diagnosis of AIP and PaCa, we analysed sera from six AIP and six PaCa patients according the strategy presented in Figure 1. For this purpose the sera were first immunodepleted of high-abundance proteins, then labelled with fluorescence dyes and separated by 2-dimensional gel electrophoresis. Statistical analysis of the obtained gel images with a DeCyder software package revealed 39 significantly changed (p<0.05) proteins between AIP and PaCa groups. The matching protein spots with the highest differential regulation (n = 14) were excised from preparative 2D-PAGE gels and subjected to in-gel tryptic digestion. Tryptic peptide mixtures were analysed with MALDI-TOF-MS and identified by a database search using the MASCOT algorithm. A list of the identified, differentially regulated proteins in AIP and PaCa is summarised in Table 2. One spot was lost during the preparation but later identified as apolipoprotein A-II. For its identification the Cy2-stained gel was subsequently silver-stained, the spot was cut out, digested with trypsin and subjected to mass spectrometry analysis.

**Table 2:** MALDI-TOF-MS.

Spot-	NCBInr	Mass	Mascot	Protein	Ratio	P value*	Seq.	pI
ID	Acc.No.	[Da]	Score		AIP/PaCa		cover [%]	
30	gi|184747	36525	87	immunoglobulin gamma-1 heavy chain constant region	2.99	0.0045	38	9.3
31	gi|184747	36525	101	immunoglobulin gamma-1 heavy chain constant region	2.45	0.0038	40	9.3
46	gi|156627579	22921	123	tetranectin	1.62	0.017	71	5.4
47	gi|178775	28944	260	proapolipoprotein A-I	3.0	0.0002	79	5.3
48	gi|156627579	22921	191	tetranectin	1.55	0.015	77	5.4
53	gi|90108664	28061	279	chain A, Crystal Structure Of Lipid-Free Apo A- I	2.9	0.01	74	5.3
56	gi|90108664	28061	270	chain A, Crystal Structure Of Lipid-Free Apo A- I	2.87	0.002	75	5.1
	gi|170684606	23523	100	immunoglobulin kappa 1 light chain			58	5.6
58	gi|223976	42344	106	haptoglobin Hp2 20 kDa N-term. fragment!	−3.81	0.023	25	6.3
59	gi|223976	42344	125	haptoglobin Hp2 20 kDa N-term. fragment!	−3.52	0.042	24	6.3
64	gi|14719497	12671	94	chain A, transthyretin Thr119met protein stabilisation	2.0	0.022	63	5.2
200	gi|671882	8708	68	apolipoprotein A-II	3.56	0.0008	67	5.1
204	gi|3337390	38722	228	haptoglobin	−2.65	0.013	58	6.1
206	gi|178849	36302	136	apolipoprotein E	−1.48	0.009	61	5.5
207	gi|3337390	38722	183	haptoglobin	−1.79	0.01	44	6.1

Mass spectrometric identification of proteins with significant different levels in sera of AIP and PaCa patients.

* ) P value: Student's *t* test, unpaired two tailed, calculated by the image analysis DeCyder 2D 6.5 software.

We identified apolipoprotein A-I, apolipoprotein A-II and transthyretin with an AIP/PaCa ratio of 3, 3.5 and 2, respectively. In AIP we also found elevated serum levels for tetranectin and immunoglobulins, whereas in PaCa sera, apolipoprotein E and haptoglobin (PaCa/AIP ratio of 1.6 and 3.8 respectively) were found elevated.

### Conformation of differential expression of candidate biomarker proteins

**Figure 3:**
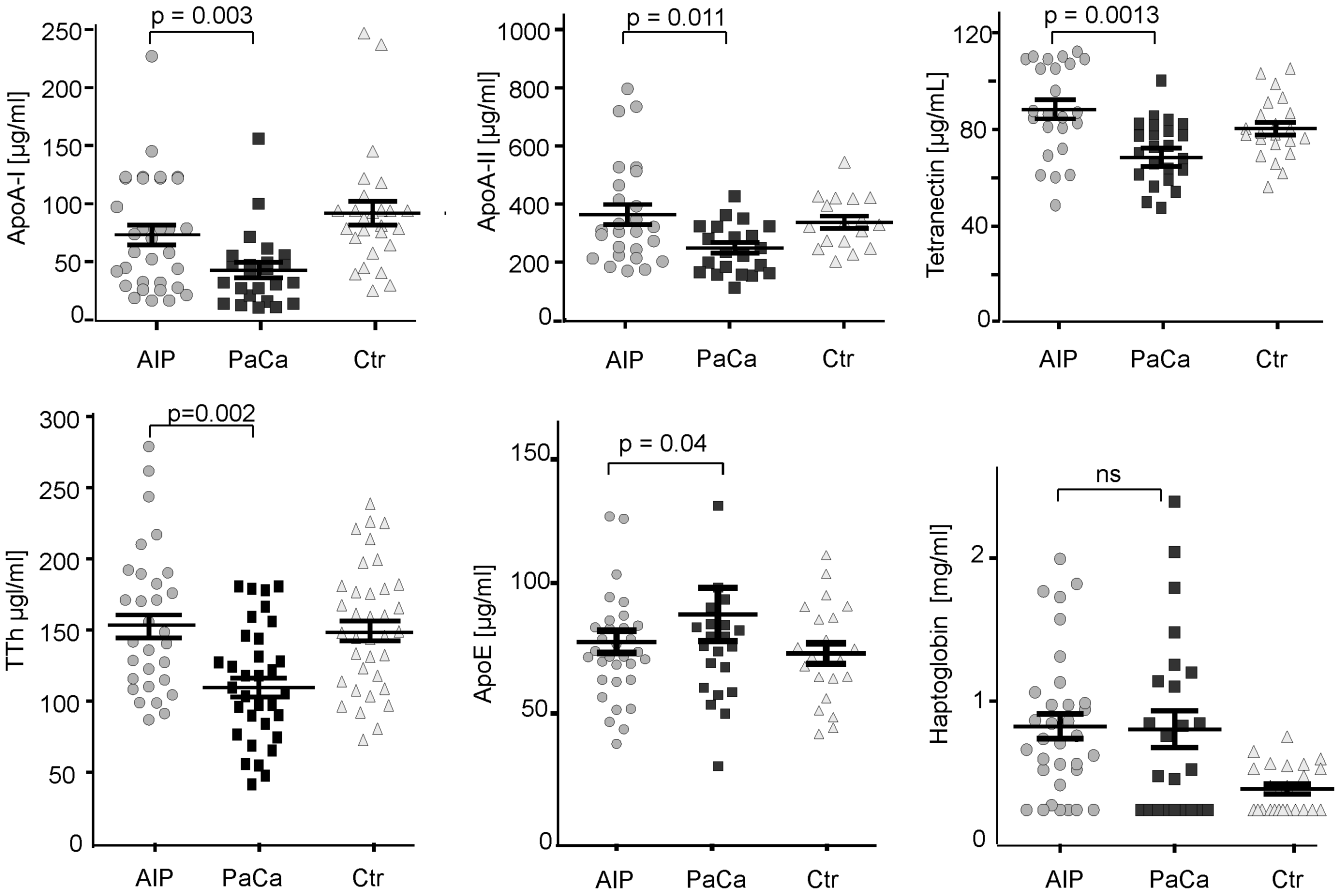
Serum levels and distribution of representative protein markers in sera from AIP-, PaCa-patients and controls assessed by ELISA. Dot plots of apolipoprotein A-I, apolipoprotein A-II, apolipoprotein E, transthyretin, tetranectin, and haptoglobin for each group. Statistical analysis was performed with GraphPad Prism5 applying Mann Whitney test (two tailed). Values are shown as a mean with SEM.

To evaluate the diagnostic accuracy of the biomarkers candidates found by 2D-DIGE and MS, we measured serum levels of apolipoprotein-A-I, apolipoprotein-A-II, apolipoprotein-E, transthyretin, tetranectin, and haptoglobin levels by commercially available ELISAs in a cohort of 32 AIP, 30 PaCa, and 30 healthy controls of randomly picked samples. With these assays we were able to discriminate the AIP sera from the PaCa group, and confirmed the mass spectrometry results. With the exception of haptoglobin (p = 0.1), all tested markers here revealed significant differences (p <0.05). Figure 3 shows the plots of the protein results for each group. Values are shown as a mean with SEM.

### Biometric analysis

**Figure 4:**
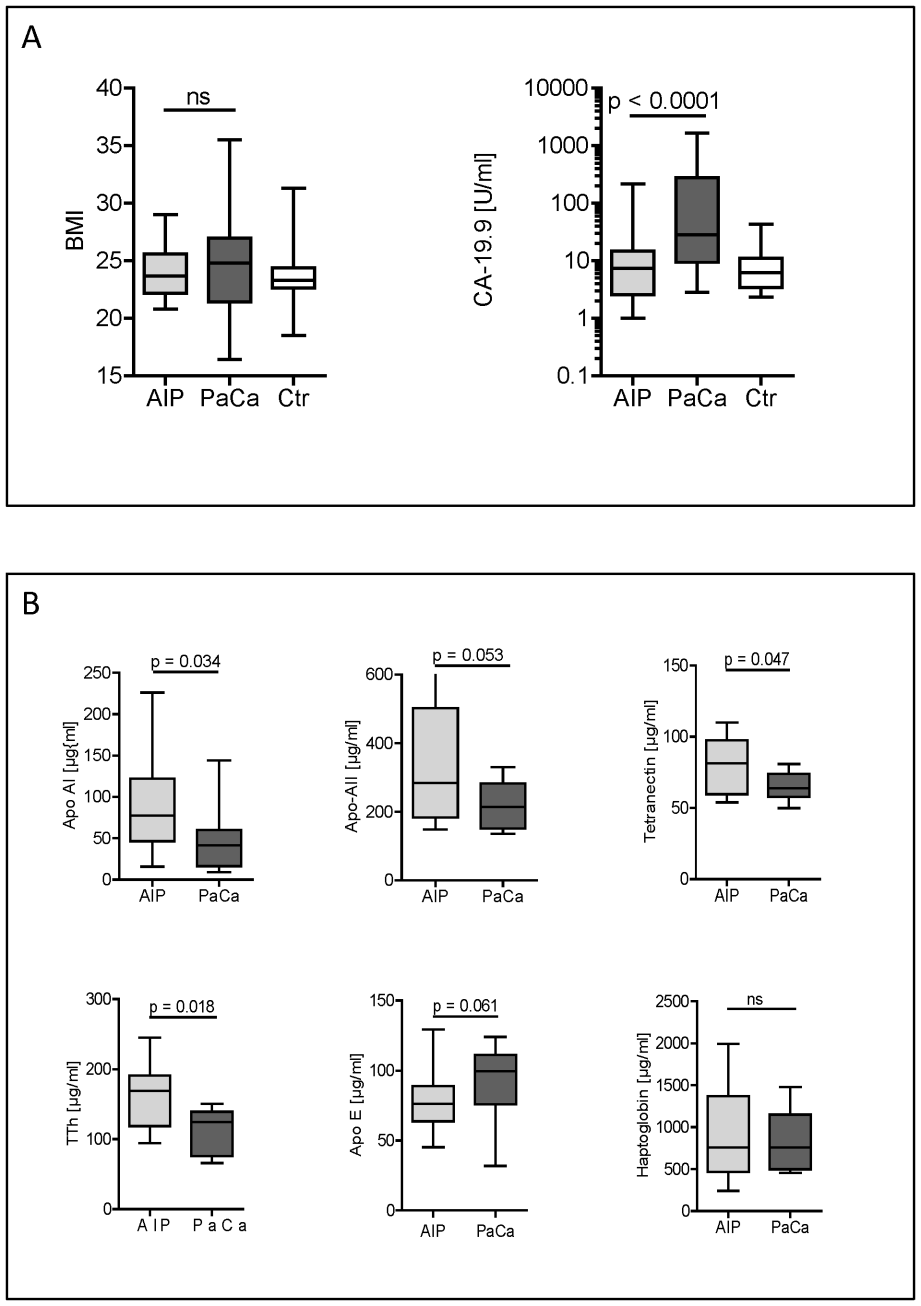
Box plot chart of BMI and of serum analysis. **A**: Body mass index and CA 19-9 levels in the AIP-, PaCa- and control-group used in the ELISA-based validations. The BMI and the CA 19-9 values of three groups AIP, PaCa, and Control are presented as box-and-whisker plots. Comparisons between the three groups were performed by using the Kruskal-Wallis test as overall test followed by the Mann-Whitney u test for pairwise comparisons. **B**: Reevaluation of the ELISAs for the six markers applying a CA 19-9 cut-off of <37 U/ml. With respect to the 6 protein markers the subgroups of AIP and PaCa defined by a CA 19-9 value <37 U/ml were compared with the one-sided Mann-Whitney u test. Significance was accepted by the 95% level. Box plots with max, Q3, median, Q1 and min.

The categorized CA 19-9 and BMI data of the three groups are presented in Table 3 and Table 4 and distribution range is shown as box-and-whisker plot in Figure 4A. To evaluate the strength of linear association between the levels of ELISA-based validations with body mass index (BMI) and levels of CA 19-9 correlation coefficients for all six protein markers were determined.

**Table 3:** CA 19-9 of the groups used for the validation of the six markers.

	CA 19-9<37 U/ml	CA 19-9>37 U/ml	
Group	n (%)	n (%)	total n
PaCa	21 (38.89)	33 (61.11)	54
AIP	16 (100)	0	16
Co	33 (100)	0	33

**Table 4:** BMI of the groups used for the validation of the six markers.

	BMI<19	BMI 19–25	BMI 26–30	BMI>30	
Group	n (%)	n (%)	n (%)	n (%)	total n
PaCa	1 (1.78)	30 (53.57)	21 (37.5)	4 (7.14)	56
AIP	0	14 (66.7)	7 (33.3)	0	21
Co	0	17 80.96)	3 (14.29)	1 (4.76)	21

In the association analysis of the six markers and BMI only tetranectin revealed for the three groups as a whole a negative correlation coefficient with a value of r = −0.374 and p = 0.01. This negative correlation was even more pronounced when the PaCa group alone was analysed with r = −0.588 and p = 0.006.

The association analysis of the six markers and CA 19-9 levels for the three groups as a whole revealed a negative correlation for apolipoprotein A-I and transthyretin with the correlation coefficients r = −0.388 (p = 0.012) and r = −0372 (p = 0.0235), respectively. Analysing PaCa alone a negative correlation coefficient was found only for apolipoprotein A-II with a value of r = −0.439; but failed the significance level with p = 0.08.

Because all AIP samples showed CA 19.9 serum levels below 37 U/ml we compared its ELISA data with the corresponding PaCa subgroup (n = 21) with CA 19-9 levels below 37 U/ml. Statistical analysis revealed still significant differences for apolipoprotein A-I, tetranectin and thransthyretin. Apolipoprotein A-II (p = 0.053) and apolipoprotein E (p = 0.061) slightly failed the significance level (Fig. 4B).

## Discussion

Distinguishing AIP from PaCa through non-invasive approaches such as serum biomarkers, or imaging is important as up to 11% of patients undergoing surgery for pancreatic cancer are diagnosed to have a benign inflammatory condition. Serologic markers such as elevated γ-globulins, particularly IgG4, were suggested as good candidates for differential diagnosis. Elevated IgG4 levels are regarded as the most sensitive serum marker for diagnosing AIP [18]. However, it has been recently shown that IgG4 can also be elevated in up to 10% of PaCa patients and is, therefore, not always specific for distinguishing AIP from PaCa [26]. Additionally, seronegative cases of AIP were described by Ghazale [27].

Our here investigated AIP study cohort showed only 58% of the samples with IgG4 values above 1.4 g/l. A variety of autoantibodies among them against nuclear antigen, lactoferrin or carbonic anhydrase II (CAII) were reported in AIP patients sera but none of them is known to be of IgG4 subclass. It is still unclear whether the formation of these autoantibodies and increased IgG4 levels play a pathogenic role or are epiphenomena of AIP [28], [29]. A research team in Japan succeeded in inducing AIP in newborn athymic mice by immunising them with lactoferrin and CAII [30]. Another research group detected antibodies against amylase α2 in AIP [31]. None of the reported markers are so specific that they can discriminate safely between AIP and PaCa.

A possible interconnection between *Helicobacter pylori* infection and AIP has been reported [19]. Several laboratories suggested a molecular mimicry as a cause, wherein antibodies directed towards *H. pylori* proteins also bind to body-own peptide sequences and induce an immune response in the pancreas [32]–[36]. Notably, in a recent work, it was shown that in 19 out of 20 screened AIP patients, autoantibodies against plasminogen-binding proteins from *H. pylori* were found, which were likely a useful serologic marker to distinguish AIP from PaCa and CP [19]. However, screening of all our AIP patients revealed an *H. pylori* seroprevalence of 43.8% and was the lowest of all groups tested. One explanation for the discrepancy between the results of the studies could be the geographically and environmentally different origin of the patients. Our results also confirm a previous study where in the pancreas of 11 AIP patients no *H pylori* urease A and 16S rDNA were detected by nested PCR [37].

Profiling of equalised and subfractionated sera from AIP and PaCa patients on ProteinChip in one of our previous studies applying the SELDI-TOF-MS technique revealed 38 potential protein markers ranging between 3.14 and 42.23 kDa [38]. To further analyse the profiling results obtained by SELDI-TOF-MS, we performed here a 2D-DIGE study on immunoaffinity depleted serum to identify marker candidates for differential diagnosis of the two diseases. Comparing the AIP and PaCa 2D-DIGE protein expression data using the DeCyder image analysis, we found in sera of AIP patients three-fold higher levels of apolipoprotein A-I, 3.5-times higher levels of apolipoprotein A-II and two-fold higher levels of transthyretin (TTh). In AIP we also found elevated tetranectin and immunoglobulins, whereas in PaCa, apolipoprotein E and haptoglobin were higher. Observations on decreased levels of apo A-I, apo A-II and TTh in PaCa and CP compared to normal individuals were made in our previous studies [17], [38], [39].

Due to the fact that the here described observations may contribute to better differential diagnostic of AIP and PaCa, we performed ELISAs for the six proteins to confirm the 2D-DIGE/MS data and to evaluate their overall performance. With the exception of haptoglobin, for which no significant difference was found, the ratios between the two groups revealed similar characteristics but the ratios were less prominent. The elevated serum apolipoprotein E levels in PaCa patients assessed in this initial study by 2D-DIGE and ELISA are in concordance with a recent study be Chen et al. Appling different approaches including ELISA they found an up-regulation of apolipoprotein E in PaCa-tissues and elevated apolipoprotein E levels in PaCa patients serum [40].

The group of AIP and control sera showed a similar distribution range for the apolipoproteins, indicating that the serum content of these proteins is comparable. The next step would be to test whether the combination of the identified markers enables us to make a reliable prediction in unknown sera samples. Also other here in the study identified proteins could be integrated in such tests. The reason for the decreased levels of the above-mentioned proteins in PaCa and CP sera, compared to healthy volunteers or AIP, is unclear but some explanations exist. Both CP and PaCa patients suffer from malnutrition and cachexia stimulating the synthesis of acute-phase proteins in the liver but the synthesis of apolipoproteins, transthyretin, and retinol-binding protein drops. However, the identification of cachexia was not possible, no dramatic weight loss in patient medical history were recorded, majority of the patients here analysed showed normal or slightly elevated BMI and only one PaCa patient had a BMI below 19 as shown in Table 4. The significant differences of the individual proteins observed in our study indicate a completely different etiology of AIP on one site and CP and PaCa on the other. However, it remains unresolved as to whether the same serum diagnostic findings are also manifested in very early stages of the disease. In this study we used sera from PaCa patients, with partly infiltrating lymph nodes, but no metastases, the most frequent stage of patients who are resected. It would be eligible to have a kind of routine screening to detect the very early PaCa stages.

The strategy to reach the less prominent proteins so called “deep proteome” through depletion of patients' sera was effective despite the fact that the significantly different serum proteins predominantly belong to the abundant protein fraction. One reason could be the posttranslational modification of the proteins. Investigations of other groups and our own studies have demonstrated that modifications and truncated forms of proteins such as apolipoproteins and transthyretin may represent disease-specific signatures [17], [41]–[43]. The modified protein forms are binding with a low affinity, or not at all, to the immobilised antibodies on the depletion columns and, therefore, are not efficiently removed. This is visible on the 2D gels as spots for the same protein but with small changes in the isoelectric point (data not shown). Simplifying the dynamic range of serum proteins by removal of the 20 most prominent proteins by immunoaffinity depletion allows detection of less prominent proteins but also has some disadvantages. It has been shown that the removal of high-abundance proteins also leads to co-depletion of low-abundance species that are carried by the depleted proteins [44].

The present results together with our earlier findings may form the basis of a combined blood test to discriminate/differentiate AIP from PaCa in the future. Such a test would be of great value particularly for patients where the CA 19-9 marker is in normal range (<37 U/ml). Evaluation of our biomarkers candidates in sera of patients with normal CA 19-9 values revealed still significant differences between AIP and PaCa for apolipoprotein A-I, tetranectin and transthyretin.

In conclusion, this study indicates that the identified proteins may have considerable potential to provide additional information for the diagnosis of AIP to choose the appropriate treatment. Further investigations are needed to validate these proteins diagnostic accuracy in lager cohorts of patients as well as to fully evaluate their clinical usefulness.
